# Preparation of Supercapacitor Carbon Electrode Materials by Low-Temperature Carbonization of High-Nitrogen-Doped Raw Materials from Food Waste

**DOI:** 10.3390/ma17163984

**Published:** 2024-08-10

**Authors:** Qingnan Mu, Chang Liu, Yao Guo, Kun Wang, Zhijie Gao, Yuhan Du, Changqing Cao, Peigao Duan, Krzysztof Kapusta

**Affiliations:** 1 Shaanxi Key Laboratory of Energy Chemical Process Intensification, School of Chemical Engineering and Technology, Xi’an Jiaotong University, Xi’an 710049, China; 202234278@mail.sdu.edu.cn (Q.M.); chang509891@stu.xjtu.edu.cn (C.L.); gy9716@stu.xjtu.edu.cn (Y.G.); kun-wang@stu.xjtu.edu.cn (K.W.); gzj0521@stu.xjtu.edu.cn (Z.G.); yhdu9901@stu.xjtu.edu.cn (Y.D.); cq.cao@mail.xjtu.edu.cn (C.C.); 2 Institute for Advanced Technology, Shandong University, Jinan 250100, China; 3 Główny Instytut Górnictwa (Central Mining Institute), Gwarków 1, 40-166 Katowice, Poland

**Keywords:** food waste, hydrothermal carbonization, pyrolysis, N-doped carbon, supercapacitor electrode

## Abstract

To address the problem of the low nitrogen (N) content of carbon materials prepared through the direct carbonization of food waste, soybean meal and egg whites with high N contents were selected to carry out carbonization experiments on food waste. At 220 °C, the effects of hydrothermal carbonization and microwave carbonization on the properties of supercapacitor electrode materials were investigated. The results show that food waste doped with soybean meal and egg whites could achieve good N doping. At a current density of 1 A·g^−1^, the specific capacitance of the doped carbon prepared by hydrothermal doping is as high as 220.00 F·g^−1^, which is much greater than that of the raw material prepared through the hydrothermal carbonization of food waste alone, indicating that the hydrothermal carbonization reactions of soybean meal, egg white, and food waste promote the electrochemical properties of the prepared carbon materials well. However, when a variety of raw materials are mixed for pyrolysis carbonization, different raw materials cannot be fully mixed in the pyrolysis process, and under the etching action of potassium hydroxide, severe local etching and local nonetching occur, resulting in a severe increase in the pore size distribution and deterioration of the electrochemical performance of the prepared carbon materials. At a current density of 1 A·g^−1^, the specific capacitance of these prepared carbon materials is 157.70 F·g^−1^, whereas it is only 62.00 F·g^−1^ at a high current density of 20 A·g^−1^. Therefore, this study suggests that the hydrothermal carbonization process is superior to the microwave pyrolysis carbonization process for preparing supercapacitor electrode materials with multiple samples doped with each other.

## 1. Introduction

Supercapacitors are considered one of the core energy storage devices used to solve the energy crisis in the 21st century because of their outstanding advantages, such as high energy density [1], fast charging and discharging rates [2], ability to release high current in a very short time, and good cycling stability [3,4]. They are commonly used in applications that require multiple rapid charge and discharge cycles rather than long-term compact energy storage, such as family cars, buses, trains, cranes, and elevators. They are used for regenerative braking, short-term energy storage, or power delivery in burst mode. Smaller capacitors can be used as memory backups for static random access memory. The electrode material significantly influences the electrochemical performance of a supercapacitor [5]. The electrode materials commonly used in supercapacitors include carbon materials, conducting polymers, metal oxides, hydroxides, etc. [6]. Among them, carbon electrode materials have been the focus of research and development because of their good conductivity, high cycle stability, low cost, etc. [7]. Owing to its high energy density, extended lifespan, cost-effectiveness in terms of raw material procurement, and broad adaptability, this technology offers several advantages [8]. Biomass-based carbon supercapacitors are currently the main substance for commercial application of supercapacitors and are expected to become the core material for energy storage today [9,10,11].

Studies have shown that the electrochemical performance of a single carbon material containing only carbon is not optimal, and the doping of carbon materials with a small number of heteroatoms can improve the electrochemical performance of the material as a supercapacitor electrode material to a certain extent [12,13]. The incorporation of heteroatoms can alter the electron distribution within carbon materials and influence their crystal structure, leading to the initiation of pseudocapacitance reactions [14], thus improving the electron transfer capacity of the material itself and enhancing its electrochemical properties [15]. The nitrogen (N) atom is a representative heteroatom, and the N in carbon materials is usually present as pyridine-N, pyrrole-N, graphite-N, or oxidized-N [16,17,18]. Shan et al. conducted doping experiments on raw cellulose by hydrothermal carbonization at 300 °C for 1 h using inorganic ammonium sulfate as the N source, and X-ray photoelectron spectroscopy tests were performed on the hydrothermal carbon obtained by hydrothermal carbonization. The results showed that N was successfully doped into the aromatic ring, and the carbon successfully doped with N atoms showed excellent electrochemical properties, indicating that the introduction of an appropriate amount of N can improve the electrochemical performance of carbon materials for supercapacitors [19]. Xu et al. conducted a study on the preparation of biocarbon for supercapacitor electrode materials through the hydrothermal carbonization of municipal sludge with a high nitrogen content, particularly from proteins present in the sludge. The high protein content was dissolved in the aqueous phase during the hydrothermal reaction. The aqueous phase served as the nitrogen source for the hydrothermal carbonization of biomass. These findings indicated the successful doping of nitrogen from the aqueous phase into the carbon material, leading to a significant enhancement in the electrochemical properties of the material [17]. Our previous study used food waste (FW) as a single raw material for direct low-temperature carbonization to prepare electrode materials with low N contents and average electrochemical properties. The selection of raw materials with high N contents and FW for cocarbonization to prepare electrode materials with high N contents is the focus of this study [20].

Soybean meal (SM), also known as soybean residue, is a solid residue left behind after the preparation of soybean oil, and its protein content is as high as 43–48%. SM is a natural renewable high-nitrogen biomass [21], and China is a large soybean-growing country. The annual production of SM accounts for approximately 30% of the global share of SM. The use of SM as a N source mixed with FW for low-temperature carbonization to prepare supercapacitor electrode materials undoubtedly provides a new path for the utilization of SM. Egg white powder (EW), a transparent gelatinous substance wrapped around the yolk, also has a high protein content. Eighty percent of the protein content in eggs comes from EW, which can contain a large amount of N-containing substances [22], so EW is also an ideal raw material for N-source adulteration. In this work, low-temperature carbonization doping experiments were conducted on FW using SM and EW as nitrogen sources to investigate whether biochar prepared from FW was successfully doped with elemental N and whether it contributed to the electrochemical performance of the prepared supercapacitor electrode materials. This research focused on the preparation of biocarbon materials for electrode materials for supercapacitors through the low-temperature carbonization of biomass FW, SM, and EW.

## 2. Materials and Methods

### 2.1. Raw Materials and Experimental Equipment

The FW was obtained from the Langyuan canteen of Iharbour Campus of Xi’an Jiaotong University (Xi’an, China) and processed according to our previous publication. The detailed information of this FW is available in Ref. [20]. SM and EW were purchased from China Textile Grain and Oil Co. and Jiangsu Zhongcheng Food Ingredients (Xuzhou, China), respectively. An OTF-1200X tube furnace was purchased from Hefei Kejing Material Technology Co., Ltd. (Hefei, China), in China for pyrolysis experiments. A CY-PY1100C-S microwave pyrolysis furnace (Hunan Huarun Microwave Technology Co., Ltd., Changsha, China) was used. A TL1200 tube furnace (Nanjing Boyuntong Instrument Technology Co., Ltd., Nanjing, China) was utilized in the activation stage. The MSG100-P5-T3-SS1-SV-R hydrothermal carbon reactor (Anhui Kemi Instrument Co., Ltd., Hefei, China) served as the reaction vessel for the hydrothermal carbonization stage.

### 2.2. FW Hydrothermal Doping Experiment

The reaction vessel for the hydrothermal admixture studies of FW, SM, and EW was a 100 mL stainless steel reactor. The reaction kettle was filled with 7.5 g of dry FW and 7.5 g of SM, and 40 mL of deionized water was added. The reaction kettle was then sealed and rinsed with argon gas for ten minutes to remove any remaining air. The reaction temperature was 220 °C, and the reaction time was 1 h. The heat source for the reaction was a salt bath. Following the completion of the reaction, the reactor underwent the same cooling procedure, and the hydrothermal carbon that was produced was dried and placed on the side. The FW and EW were mixed via the same steps used for the hydrothermal experiments; 7.5 g of FW and 7.5 g of EW were mixed well and added to the reaction kettle, and 40 mL of deionized water was added at 220 °C for 1 h to prepare the hydrothermal carbon drying mixture. Hydrothermal carbonization was performed by adding 5 g of FW, 5 g of SM, 5 g of EW, and 40 mL of deionized water at 220 °C for 1 h. SM and EW (15 g) and SM and EM (7.5 g each) were used separately for the hydrothermal carbonization experiment as a blank control. The hydrothermal carbonization methods of the SM; hydrothermal carbonization of EW; hydrothermal carbonization of FW and SM; hydrothermal carbonization of EW and SM; hydrothermal carbonization of FW and EM; and hydrothermal carbonization of FW, SM, and EW were named HS, HE, HFS, HSE, HFE, and HFSE, respectively(Xi’an, China).

### 2.3. FW Pyrolysis Doping Experiment

Doping experiments between the microwave pyrolysis of FW, SM, and EW were used to investigate the differences in electrochemical properties between the microwave pyrolysis of carbon and hydrothermal carbon for the preparation of supercapacitor electrode materials (Xi’an, China). The raw materials for the pyrolysis experiments were selected in the same way as those for the hydrothermal experiments, with a mass ratio of 1:1:1, FW to SM and EW, and a total weight of 15 g. The pyrolysis temperature was 220 °C, and the pyrolysis time was 1 h. The same argon flushing was used for 10 min to remove the air from the pyrolysis oven. The pyrolytically doped carbon obtained by microwave pyrolysis was named MFSE.

### 2.4. Preparation of Supercapacitor Electrode Materials with Doped Carbon

KOH was used to activate the doped carbons HS, HE, HFS, HSE, HFE, HFSE, and MFSE to produce pores at high temperatures. One gram of dried doped carbon was weighed, added to a 1.5% weight KOH solution and left overnight. After filtering, the material that had been filtered was removed, and the mixture was kept in an oven at 105 °C for more than 12 h. The materials that were dried are put in a tube furnace to create holes at a high temperature. The tube furnace functioned within an Ar atmosphere at a flow rate of 30 mL/min. Before removal, the temperature was increased to 800 °C at a rate of 20 °C per minute, followed by a one-hour hold at this temperature. Before utilization, the samples were rinsed with deionized water and subsequently dried in an oven at 105 °C for more than ten hours. The samples eliminated were AHS, AHE, AHSE, AHFE, AHFSE, and AMFSE. The mechanism by which KOH activated the carbon materials and increased their porosity can be seen in Ref. [23]. Combined with the experimental data and theoretical calculations, the current general view is that the redox reaction between KOH and the carbon source occurs between 400 °C and 600 °C.

2C + 6KOH → 2K + 2K_2_CO_3_ + 3H_2_ (1)

In this process, micropores are generated. When the temperature reaches 600 °C, the KOH reaction is essentially complete, and the generated K_2_CO_3_ decomposes to produce CO_2_, which further activates carbon materials through physical activation:

K_2_C → K_2_O +CO_2_ (2)

CO + C → 2CO(3)

When the temperature exceeds 700 °C, potassium compounds (K_2_CO_3_, K_2_O) are reduced by carbon to produce potassium vapor in the elemental state:

K_2_CO_3_ + 2C → 2K +3CO (4)

C + K_2_O → 2K + CO (5)

On the basis of the above discussion, three main processes exist for the KOH activation of carbon materials: (1) Reactions (1), (4), and (5) are chemical activation processes in which carbon and different compounds undergo reduction reactions to form porous network structures. (2) At high temperatures, H_2_O and CO_2_ can vaporize carbon atoms to improve the porosity of porous materials, which is a physical activation process. (3) Metal potassium can be effectively inserted into the carbon layer, leading to the expansion of the carbon layer; this expansion is irreversible and will not return to the state before intercalation because of the removal of potassium. Therefore, KOH activation is the synergistic result of chemical activation, physical activation, and carbon expansion.

A total of 167 μL of polytetrafluoroethylene, 3.75 mg of acetylene black, and 20 mg of activated material were placed in a beaker with the right amount of ethanol solution. After ten minutes of ultrasonic treatment, the mixture was placed in an oven at 105 °C for two hours, after which it was removed. The material was dried and then pressed into 10 mm diameter circular sheets. Two 15 mm diameter nickel foam disks and a 12 cm long metal strip were placed between the disks. The sample was formed into an electrode sheet for testing by applying pressure between 2 and 4 MPa.

### 2.5. Characterization of the Activated Doped Carbon

#### 2.5.1. Physicochemical Properties

An elemental analyzer (Vario EL cube CHNS/O, Elementar, Langenselbold, Germany) was used to determine the elemental compositions of the food waste, doped carbon, and activated doped carbon. X-ray photoelectron spectroscopy (XPS, Thermo ESCALAB 250XI, Thermo Fisher Scientific, Waltham, MA, USA) was used to identify the organic C and N functional groups. The morphology of the samples was examined by scanning electron microscopy (SEM, Gemini SEM 500, Carl Zeiss AG, Oberkochen, Germany). Using a JW-BK100 apparatus (Beijing JWGB Instruments Co., Ltd., Beijing, China), the specific surface area (SSA) and pore size distribution of the AHCs were ascertained via N_2_ adsorption‒desorption isotherms.

#### 2.5.2. Electrochemistry and Product Yield Calculations

An electrochemical workstation CHI1760E-B18258 was used to test the electrochemical performance of the electrode materials. The test materials were examined via electrochemical impedance spectroscopy (EIS), current charge and discharge (GCD), and cyclic voltammetry (CV). The CV and GCD test range was −1 to 0 V. The IMP had a test range of 100 kHz to 0.01 Hz. Detailed information on the electrochemical performance testing of the electrodes can be found in Ref. [24].

Gas bags were used to collect the gas products during hydrothermal doping studies, and the difference in reactor mass before and after the gas was released from the reactor was used to compute the gas yield: Y_gas_ = (m_gas_/m_feedstock_) × 100%. The liquid and solid phases of the reaction mixture were separated by vacuum filtering, and the solid-phase doped carbon was dried for 24 h at 105 °C to produce the following solid product: Y_soild_ = (m_soild_/m_feedstock_) × 100%. The water-soluble product (WSP) was obtained by weighing the separated produced liquid phase: Y_WSP_ = [(m_WSP_ − m_H2O_)/m_feedstock_] × 100%.

## 3. Results and Discussion

### 3.1. Distribution of Hydrothermal Product Yields of FW, SM, and EW

**Figure 1:**
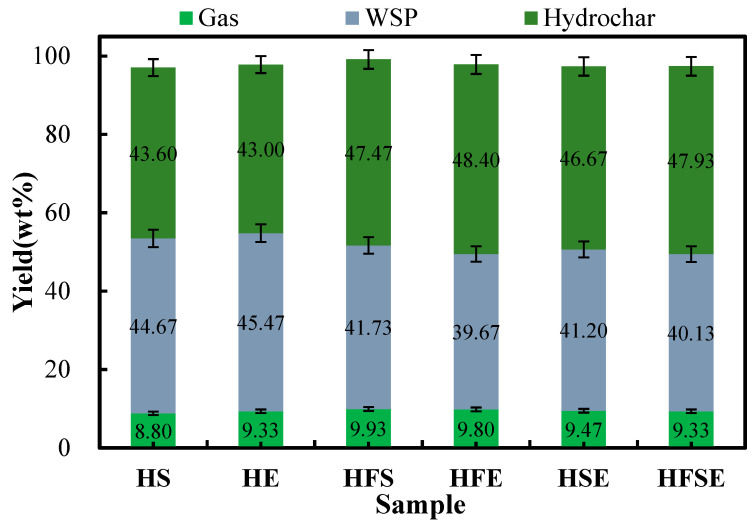
Distribution of yields of doped hydrothermal carbonization products.

The product yield distributions of each phase of the hydrothermal carbonization reactions of SM, EW, FW + SM, FW + EW, SM + EW + FW, and SM + EW produced at 220 °C for 1 h are shown in Figure 1. Three products, hydrochar, water-soluble product (WSP), and gas, were produced when each material was subjected to the hydrothermal carbonization process. Among the three products, the gas yield was the lowest regardless of the type of raw material, which does not exceed 10 wt.% and varied between 8.80 wt.% and 9.93 wt.%, because low temperature is not conducive to the generation of gas products. When SM and EW powders were hydrothermally carbonized alone, the yield of WSP was greater than that of hydrochar. However, when SM and EW powders were hydrothermally carbonized together with FW, the yield of hydrochar was greater than that of WSP. The main reason for this phenomenon is that the Maillard reaction occurs between amino acids obtained from protein hydrolysis and reduced polysaccharides obtained from cellulose hydrolysis, resulting in an increased yield of the final hydrochar. This reaction also doped nitrogen from protein into the final hydrochar. A previous study [20] suggested that the hydrothermal carbonization of FW occurred at 220 °C, and the yield of hydrochar was 57.80 wt.%, which was much greater than that of WSPE (31.40 wt.%), as FW contains both protein and cellulose polysaccharides.

### 3.2. Carbon Doping and Activated Carbon Doping Element Analysis

Elemental tests were performed on SM and EW raw materials, hydrothermally doped carbon, activated hydrothermally doped carbon, and activated pyrolytic carbon via elemental analyzers, and the results are shown in Table 1. The elemental N contents of SM and EW far exceeded the elemental N content of FW, with the elemental N content in SM being approximately three times greater than that in FW. Since temperature is the primary factor affecting the degree of the hydrothermal carbonization of biomass, the content of hydrothermally doped carbon C was primarily distributed between 64 and 67 weight percent. The contents of H, N, S, and O did not significantly differ. This suggests that for similar experimental raw materials at the same temperature, the degree of carbonization was not significantly different. The higher elemental N content of hydrothermally doped carbon after activation indicates a significant doping effect.

During the activation phase, the carbon material further carbonizes, as seen primarily by the decrease in O and decrease in H to increase in C. The reaction of sample AHSE to increase C and decrease O is more significant, with the highest C content of 79.13%, and the other samples have similar C and O contents. The N content of hydrothermally doped carbon becomes slightly lower than that of hydrothermally doped carbon. The microwave pyrolysis of activated carbon AMFSE had the lowest C content and the highest O content, indicating that the microwave carbonization process is not as good as the hydrothermal carbonization process for increasing the C de O reaction, and the N content is also lower than that of the activated hydrothermally doped carbon, mainly because the pyrolysis processes between the raw materials do not react with each other; additionally, the pyrolysis processes between each other independently result in an uneven distribution of products, and the doping effect is not obvious, which easily leads to the emergence of local differences in the overall phenomenon of the material, so that the material properties vary greatly.

### 3.3. Carbon Doping and Activated Carbon Doping Morphology and Structural Characterization

**Figure 2:**
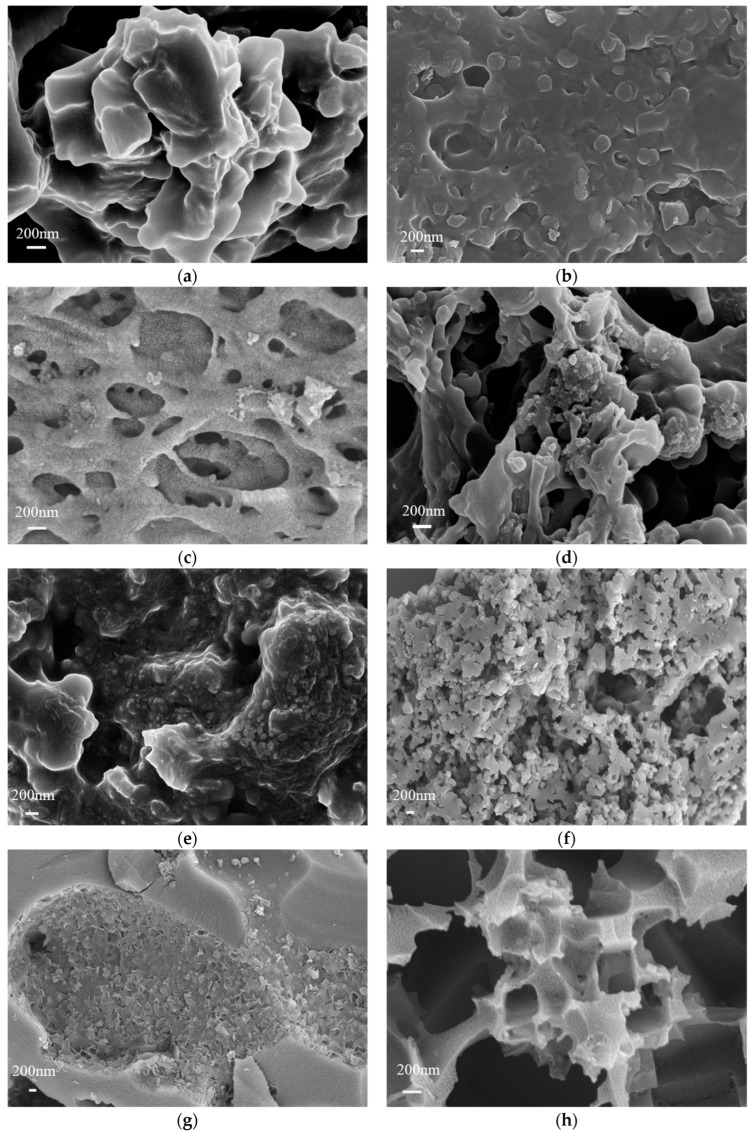
SEM images of (**a**) HFS; (**b**) AHFS; (**c**) HFE; (**d**) AHFE; (**e**) HFSE; (**f**) AHFSE; (**g**) MFSE; and (**h**) and AMFSE.

Figure 2 displays SEM images of normal hydrothermally doped carbon, active hydrothermally doped carbon, pyrolytically doped carbon, and activated pyrolytically doped carbon. The hydrothermal carbon of the FW-doped SM is displayed in Figure 2a,b both before and after activation. There was a dense layer on the surface with no discernible pore structure, indicating the limited specific surface area of the HFS. The incorporation of SM did not appear to facilitate the etching process or the formation of pores by KOH. The accumulation phenomenon on the surface of the activated AHFS was significantly greater than that on the surface of the HFS. In contrast, there was no corresponding accumulation on the surface of the hydrothermal carbon activation of the FW-doped EW before and after activation. The HFE surface was distributed in a rod-like structure with an obvious pore structure, and the activated AHFE was mainly microporous and macroporous, indicating that the EW was easily etched by KOH under high-temperature activation conditions to produce a complex pore structure [25]. Figure 2e,g show the hydrothermal carbon and microwave pyrolysis carbon of three raw materials, namely, restaurant waste, SM, and EW, mixed with each other, respectively.

The surface morphology of MFSE differs noticeably from that of HFSE, and Figure 2g shows that the middle part of MFSE has more pyrolysis than the edge does, resulting in the appearance of a depression structure. This phenomenon is primarily caused by the doped materials not interacting or reacting with one another during the microwave pyrolysis reaction, which causes the easy-to-pyrolyze components to pyrolyze first and leave the harder-to-pyrolyze parts mostly behind [26]. SM is clearly less likely to undergo pyrolysis than FW and EW are. Therefore, the degree of pyrolytic carbonization is low, which cannot ensure the simultaneous and uniform pyrolysis of the material, which has a great negative impact on the electrochemical properties of the material [27]. The activated FW-, SM-, and EW-doped carbon surfaces nevertheless have very different surface morphologies, as shown in Figure 2f,h. In contrast to the AMFSE surface, which has numerous square deep grooves with side lengths longer than 200 nm, the AHFSE surface is equally distributed, has more pore structures and is largely microporous. The main cause of this problem is that throughout the pyrolysis process, the various raw ingredients are not thoroughly combined and integrated. This issue primarily arises from the incomplete combination and fusion of diverse raw ingredients during the pyrolysis process. The use of KOH results in an etching effect that induces a pronounced local etching phenomenon, leading to a significant nonuniform distribution of pore sizes. Moreover, the presence of large and nonporous local features substantially diminishes the efficiency of electron transfer in the material’s charge and discharge processes, and it also diminishes the suitability of the material as an effective host for the active material, all of which negatively impact the material’s electrochemical properties. In summary, for low-temperature carbonization experiments involving a variety of raw materials, the hydrothermal carbonization process is superior to the microwave carbonization process. Hydrothermal carbonization promotes mutual reactions between materials, resulting in the fusion of carbon materials, whereas pyrolytic carbonization fails to facilitate material interactions, leading to uneven material pyrolysis and significant structural differences in the carbon materials obtained [7].

**Figure 3:**
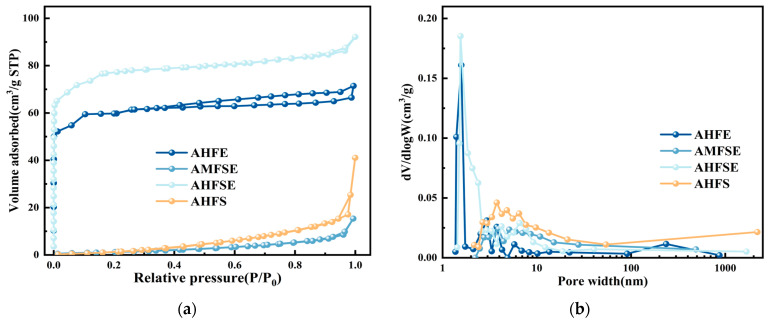
BET test results for AHFE, AMFSE, AHFSE, and AHFS. (**a**). N_2_ adsorption/desorption curve; (**b**). Pore size distribution curve.

Figure 3 shows the isotherms and pore size distributions of the nitrogen elution adsorption of the AHFE, AMFSE, AHFSE, and AHFS samples. AHFE and AHFSE show typical type I and type IV composite curve distributions, and both show a vertical increase in N_2_ adsorption at P/P_0_ < 0.1, indicating the existence of many microporous structures inside the materials and that the KOH solution has good performance in the formation of pore throats at high temperatures, which is consistent with the SEM plot results. At a relative pressure in the range of 0.1–0.9, the sorption and desorption isotherms of nitrogen in AHFE and AHFSE show a slowly increasing trend, indicating the existence of trace mesoporous structures in the materials [28], and H_4_-type hysteresis loops appeared larger in AHFE than in AHFSE, i.e., the number of mesopores in sample AHFE was greater than that in sample AHFSE. At a relative pressure > 0.9, the isotherms of nitrogen for AHFE and AHFSE slowly increased, indicating the existence of some mesoporous structures in the materials [29].

With increasing relative pressure, the sorption and desorption isotherms of nitrogen increase indicate the existence of certain macroporous structures in the material. The pore size distribution of the materials in Figure 3b indicates that the predominant pore structures present are micropores. Specifically, the AHFE and AHFSE micropores are predominantly distributed within the pore size range of 1–2 nm. The isotherm shape of the nitrogen elution adsorption of the AMFSE and AHFS samples is a type III curve distribution, and the adsorption of N_2_ is negligible when the relative pressure is between 0 and 0.05, indicating that the proportion of micropores contained inside the materials is extremely low or does not contain micropores [10], which is consistent with the SEM surface morphology structure of AMFSE and AHFS. Figure 3b shows that AMFSE and AHFS have almost no data in the pore size range of 1–2 nm, once again demonstrating the low microporous content of both materials. Within the relative pressure range between 0.10 and 0.90, the N_2_ adsorption of AMFSE and AHFS gradually increased with increasing relative pressure. The nitrogen adsorption and desorption isotherms of AFSE consistently exceeded those of AMFSE, indicating the presence of certain mesopores in the materials. Additionally, the number of mesopores in AHSE was greater than that in AMFSE, a result well supported by their pore size distribution, as shown in Figure 3b. The pore structures of AMFSE and AHFS consist mainly of mesopores with pore sizes ranging from 2 to 50 nm. At a relative pressure > 0.9, the nitrogen elution isotherm of AHFS was significantly greater than those of AHFE, AMFSE, and AHFSE. This discovery indicates the prevalence of macropores, which are defined by pore structures exceeding 50 nm in size. This finding aligns with the findings of the pore size distribution analysis illustrated in Figure 3b. The pore structures of AMFSE and AHFS are mainly mesopores with pore sizes between 2 and 50 nm. At a relative pressure of P/P_0_ > 0.9, the nitrogen elution isotherm of AHFS increased more significantly than those of AHFE, AMFSE and AHFSE did, indicating the presence of the most macropores, which were mainly pore structures with pore sizes larger than 50 nm, in agreement with the results of the pore size distribution plot in Figure 3b.

Table 2 presents the pore structure parameters of the AHFE, AMFSE, AHFSE, and AHFS samples. The BET-specific surface areas of AHFE and AHFSE were 207.8 m^2^/g and 268.6 m^2^/g, respectively. Specifically, the specific surface areas of the micropores were 179.8 m^2^/g and 248.2 m^2^/g, accounting for 86.52% and 92.4%, respectively. The porosity of the materials was measured to be 92.4%, and the average pore sizes were 2.20 nm and 2.38 nm, indicating a predominant distribution of micropores.

**Figure 4:**
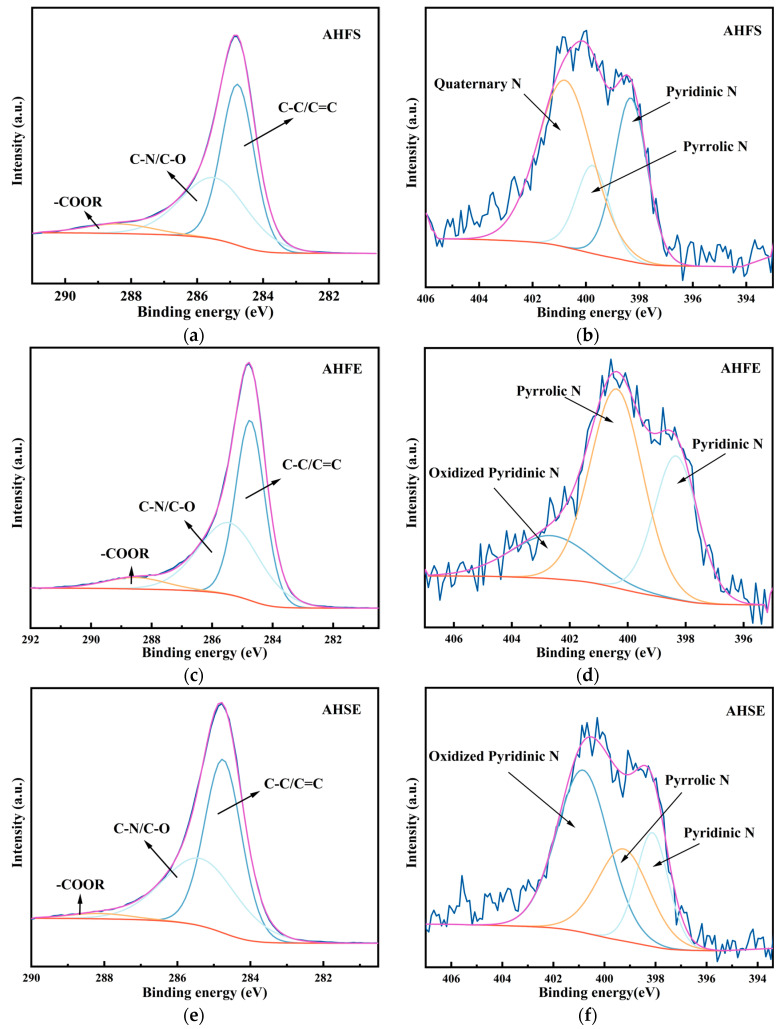
Binding energy profiles for AHFS, AHFE, and AHSE: (**a**,**b**) C1s and N1s spectra of AHFS; (**c**,**d**) C1s and N1s of AHFEs; (**e**,**f**) C1s and N1s of the AHSE.

**Figure 5:**
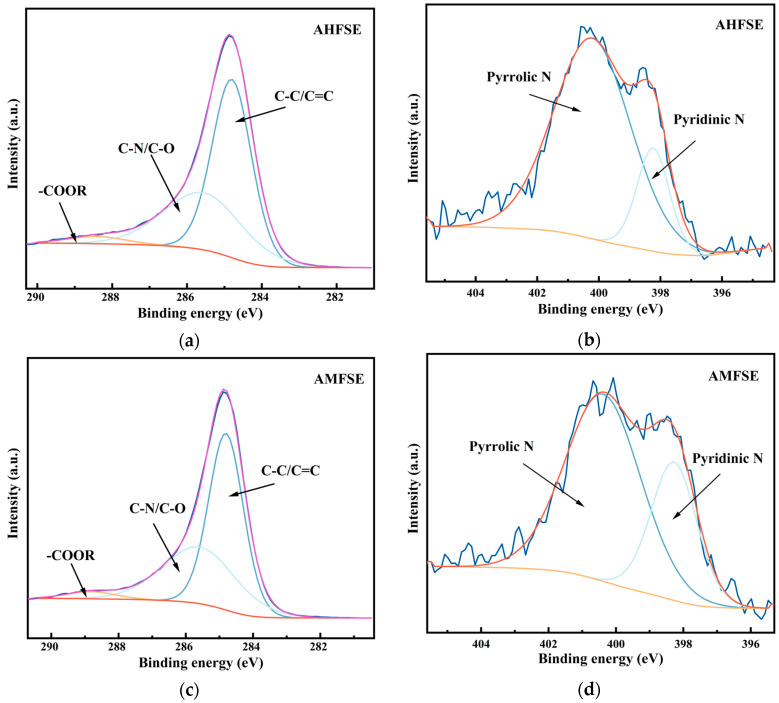
Binding energy profiles for AHFSE and AMFSE: (**a**,**b**) C1s and N1s of AHFSE; (**c**,**d**) C1s and N1s of AMFSE.

The XPS spectra of the activated hydrothermally doped carbon and microwave pyrolysis-doped carbon are shown in Figure 4 and Figure 5. Compared with those of the FW, the intensities of the N1s peaks of the activated carbon obtained through the direct hydrothermal carbonization of the FW and the activated carbon obtained by pyrolysis carbonization were significantly greater, indicating that the low-temperature carbonization of SM and EW together with FW promoted good N doping. Figure 4a,c,e shows the C1s spectra of AHFS, AHFE, and AHSE, respectively. The C1s spectra of all three samples contain three identical characteristic peaks, C-C/C=C, with a binding energy of 284.8 eV. Figure 4 also shows the XPS spectra of hydrothermally carbonized activated carbons from food waste, soybean meal, and egg white powder doped with each other [30]. The highest content of C‒C/C=C peaks indicates that the C in the carbon materials obtained from the hydrothermal doping experiments still exists mainly in the form of C‒C and C=C, and the carbon skeleton formed by carbon‒carbon bonds is the basic framework of the materials. The carbon skeleton is the basic framework of the material. The content of the functional group C-N/C-O was the second highest, but the areas of its C-N/C-O peaks all exceeded the area of the C-N/C-O peaks in the carbonization of the FW alone, indicating that the high protein content of SM and EW improved the N-doping effect. Compared with the C=O functional group, the carbonization of the FW alone did not appear, but the -COOR ester functional group appeared, but its content was very low, and the intensity of the peak was the weakest. This effect was caused primarily by the esters present in both SM and EW, which were converted during the cohydrothermal carbonization experiment from an unstable C=O functional group to a stable ester-COOR functional group.

Figure 4b,d,f show the N1s spectra of AHFS, AHFE, and AHSE, respectively. The distinctive peaks observed in the N1s spectra of the three samples exhibit significant variations. Specifically, the N1s spectra of AHFS and AHSE are characterized primarily by pyridine-N, which is evident from a prominent peak at a binding energy of 398.3 eV [31], pyrrole-N with a binding energy of approximately 399.8 eV [32], and graphite-N with a binding energy of approximately 400.8 eV [33]. The impact of pyridine-N and pyrrole-N on the electrochemical characteristics of materials varies [34]. The incorporation of lone pairs of electrons through pyridine-N facilitates the creation of additional electrochemically active sites in the materials. This phenomenon enhances the Faraday capacitance, thereby enhancing the electrochemical properties of the materials [16]. On the other hand, pyrrole-N demonstrates enhanced charge mobility and serves as an effective electron donor [35]. In contrast to those of both AHFS and AHSE, which have the typical peaks of pyridine-N, pyrrole-N, and pyridine-N-oxide, the characteristic peaks of the N1s peak of AHFE are very different [36]. The sequence of the distinctive peak intensities is pyrrole-N, pyridine-N, and pyridine-N-oxide, with pyrrole-N of AHFE having the highest intensity. Among the three samples, the pyrrole-N characteristic peak of AHFE had the highest intensity. During the material’s charge/discharge process, pyridine-N-oxide can effectively facilitate electron transport, hence enhancing the material’s electrochemical characteristics [37].

The C1s and N1s spectra of activated doped hydrothermal carbon AHFSE and activated doped microwave pyrolysis carbon AMFSE obtained from hydrothermal carbonization and microwave carbonization experiments with the same mass ratios of FW, SM, and EW are shown in Figure 5a–d, where the FW, SM, and EW are doped with each other and their two dopants. The three characteristic peaks present in the C1s spectrum of activated doped hydrothermal carbon are attributed to C-C/C=C, C-N/C-O, and -COOR. Additionally, the ratio of the three peaks is dominated by C-C/C=C, followed by C-N/C-O and -COOR. The lowest content indicates that AHFSE and AMFSE are also based on the carbon skeleton formed by carbon‒carbon bonds as the basic framework of the materials, and they are both typical carbon materials. The C-N/C-O and -COOR characteristic peaks of C1s of AHFSE and AMFSE were slightly greater than those of AHFS, AHFE, and AHSE, indicating that the simultaneous addition of SM and EW was favorable for the formation of C-N/C-O and -COOR.

Figure 5b,d show the N1s spectra of AHFSE and AMFSE, respectively. Compared with those of the N1s spectra of AHFS, AHFE, and AHSE, the intensities of the N1s peaks of both of them increase, indicating that the mutual doping among the three has a mutual promotion effect on the introduction of N, but the characteristic peaks of the N 1s spectra of AHFSE and AMFSE are very different from those of AHFS, AHFE, and AHSE. The N1s spectra of AHFS, AHFE, and AHSE are substantially different from those of AHFSE and AMFSE, which only have two prominent peaks: graphite-N and pyridine-N. Graphite-N can markedly improve the capacitive performance of a material by increasing the conductivity of carbon-based electrodes during the charging and discharging processes and, to a lesser extent, by reducing the electron transfer resistance during these operations [38,39].

### 3.4. Electrochemical Performance of Doped Carbon

#### 3.4.1. Electrochemical Performance of the AHS and AHE

**Figure 6:**
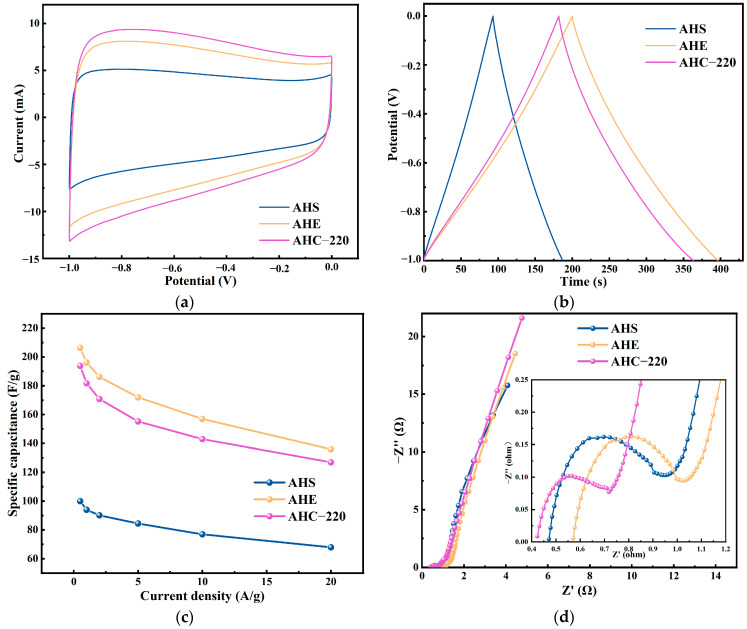
Electrochemical properties of the AHS and AHE. (**a**) CV curves of AHS, AHE, and AHC−220. (**b**) GCD curves of AHS, AHE, and AHC−220. (**c**) Specific capacitance curves at different current densities. (**d**) Nyquist impedance plots.

The electrochemical characteristics of the electrodes formed from the AHS and AHE samples were tested via a three-electrode test system. The test findings were compared with those of sample AHC-220, which was made by hydrothermally carbonizing FW alone at 220 °C. CV tests, GCD tests, and EIS tests were used to assess the electrochemical characteristics of the materials. The CV curves of the AHS and AHE, compared with those of sample AHC-220, are displayed in Figure 6a at 10 mV/s. The CV curves of both the AHS and AHE samples have rectangular-like shapes similar to those of the AHC-220 sample, and both show good and similar quasirectangular shapes, indicating that during the cyclic voltametric curve measurements, the electrolyte ions exhibit a faster ion response [40], and the quasirectangular shape distribution indicates that both have good bilayer capacitance behavior. At approximately −0.8 V for the AHE and AHC-220, slight protrusions and distortions were detected. This observation suggested that the redox reaction of the oxygen-containing functional groups on the AHE sample surface, which occurred during rapid charge and discharge cycles, resulted in pseudocapacitance [41]. The CV curve of sample AHS near −0.8 V is a horizontal straight line, indicating that no significant redox reaction occurred on its surface during cyclic voltammetry testing. To some extent, the electrochemical characteristics of a sample can be reflected in the integrated area of the closed CV curve. The GCD curves of the AHS and AHE samples are displayed in Figure 6b in comparison to that of sample AHC-220 at 1 A/g. The charge/discharge time of the AHE is marginally longer than that of sample AHC-220, but the charge/discharge time of sample AHS is much shorter at the same current density. These findings suggest that among the supercapacitor electrode materials, soybean meal from the activated hydrothermal carbon AHS—which was prepared by hydrothermal carbonization at 220 °C—had the worst energy storage capacity, which is consistent with the CV curve results shown in Figure 6a. The constant current charge and discharge curves of the AHS and AHE materials obtained through individual hydrothermal carbonization processes of SM and EW at 220 °C exhibit shapes identical to those of sample AHC-220. They display symmetrical triangle shapes and demonstrate similar charge and discharge durations [42,43]. Figure 6c compares the specific capacitance curves of the AHS and AHE samples with that of sample AHC-220. The specific capacitance of sample AHS is significantly smaller than that of sample AHC-220, and the specific capacitance of sample AHE is larger than that of sample AHC-220 at different current densities. When SM was selected as the raw material to prepare the supercapacitor carbon material, the resulting carbon material showed the lowest electrochemical performance [44]. The specific capacitance of the sample AHE is the highest at the equivalent current density, suggesting superior energy storage capacity. Additionally, the rate of change in the specific capacity gradually decreases with increasing current density. The specific capacitance of the AHE remains consistent at 136.0 F/g even when subjected to a high current density of 20 A/g, whereas it is 196.2 F/g at 1 A/g. At elevated potentials, the capacitance retention rate is 69.32%, which is slightly lower than that of sample AHC-220. The AHS sample has a specific capacitance of 94.0 F/g. At high potentials, the capacitance retention of the AHS sample is 72.34%, which is somewhat greater than that of the AHC-220 sample at high potentials. Figure 6d shows the impedance diagrams of the AHS and AHE samples compared with that of the AHC-220 sample. The charge transfer resistances (R_ct_) of the AHS and AHE samples during charging and discharging are essentially the same and greater than that of the AHC-220 sample, indicating that both have higher energy consumption with higher internal resistance during charging and discharging [45]. The intercept of the semicircle with the horizontal axis represents the equivalent series resistance (R_s_) generated in the working electrode [46]. Both the AHS sample and the AHE sample exhibit near-linear behavior in the low-frequency range, confirming that the activated hydrothermal carbon made from SM and EW at 220 °C for supercapacitor electrode materials both exhibit good double-layer capacitive behavior. Additionally, the electrochemical performance of the AHE sample is substantially superior to that of the AHS sample.

#### 3.4.2. Electrochemical Performance of AHFS, AHFE, AHSE, and AHFSE

**Figure 7:**
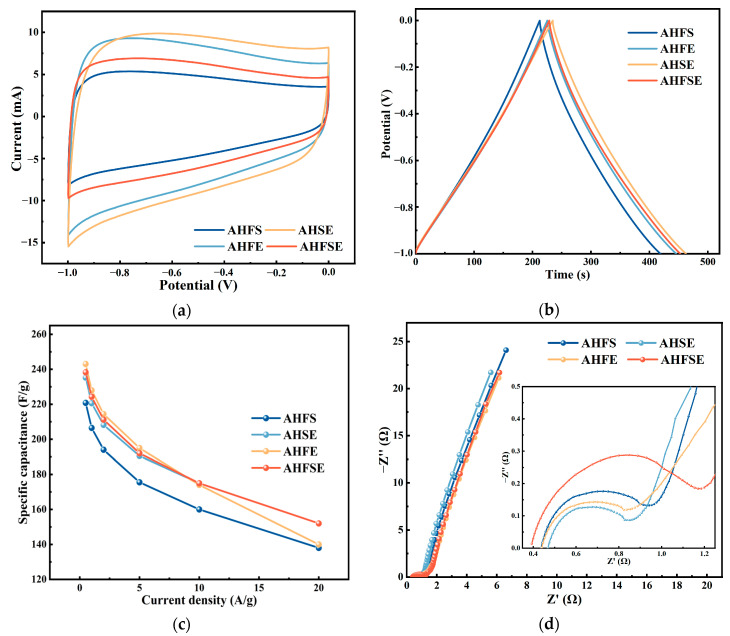
Electrochemical properties of AHFS, AHFE, AHSE, and AHFSE. (**a**) CV curves obtained at 10 mV/s. (**b**) GCD curves obtained at 1 A/g. (**c**) Specific capacitance curves obtained at different current densities. (**d**) Nyquist impedance plots.

The electrochemical characteristics of the materials were assessed via CV curves, GCD curves, specific capacitance values, and EIS curves. The same three-electrode test equipment was used to examine the electrochemical properties of the four types of doped activated carbon materials. With a scan rate of 10 mV/s, the CV curves of the four types of doped activated carbon materials are displayed in Figure 7a. Each curve exhibits a good quasirectangular shape that is consistent with the acceptable bilayer capacitance performance [47]. The four types of doped activated carbon materials showed no obvious concave or convex deformation near −0.8 V and presented a horizontal straight line shape distribution, indicating that the electrode materials prepared through the hydrothermal carbonization of FW-doped SM and EW showed improved surface redox reactions during cyclic voltammetry testing. There were significant differences in the integrated areas of the CV curves of the samples at a scan rate of 10 mV/s, and the integrated areas of the curves were in the order of largest to smallest for the AHSE, AHFE, AHFSE, and AHFS samples. The GCD curves of AHFS, AHFE, AHSE, and AHFSE at 1 A/g are displayed in Figure 7b. One cycle’s charge and discharge periods at 1 A/g were more than 400 s, and these times were longer than those of supercapacitor electrode materials achieved only through the hydrothermal carbonization of FW, SM, and EW under the same conditions. The GCD curves of the samples were symmetrical. Each sample exhibited a symmetric triangular distribution in its GCD curve, suggesting strong electrochemical reversibility and bilayer capacitive behavior. Figure 7c shows the specific capacitance curves of the AHFS, AHFE, AHSE, and AHFSE samples at different current densities. At various current densities, the specific capacitance of the AHSE sample is slightly greater than that of the AHFE and AHFSE samples and significantly greater than that of the AHFS sample at high current densities. This pattern changed at 20 A/g, and the specific capacitance of the AHFE and AHFS samples exceeded that of the AHSE sample, indicating that the AHSE sample has a high electrical energy capacity at low current density, but its capacitance retention at high potentials is low. The AHSE sample had a specific capacitance of 228.0 F/g at a current density of 1 A/g and only a 61.40% capacitance retention at high potentials. The samples made from activated hydrothermal carbon in the hydrothermal doping experiments all had specific capacitances that were greater than those of the samples made from EW, SM, and FW alone. This suggests that the mutual doping of the three materials has a positive effect on the materials’ electrochemical characteristics.

Figure 7d shows impedance diagrams of the AHFS, AHFE, AHSE, and AHFSE samples. The R_ct_ and R_s_ values of the AHFS, AHFE, and AHFSE samples are basically the same during the charging and discharging process. The equivalent R_s_ of sample AHSE is slightly smaller than that of the other samples, but its R_ct_ is much larger than that of the other samples, which is approximately twice as large as that of the other samples. The higher the charge transfer resistance of the material at high current densities, the greater the impedance to electron transfer during the charging and discharging processes of the material. This impedance is primarily manifested in a more pronounced reduction in the specific capacitance of the material at elevated potentials. The primary factor contributing to the decreased capacitance retention of AHSEs at elevated potentials.

**Figure 8:**
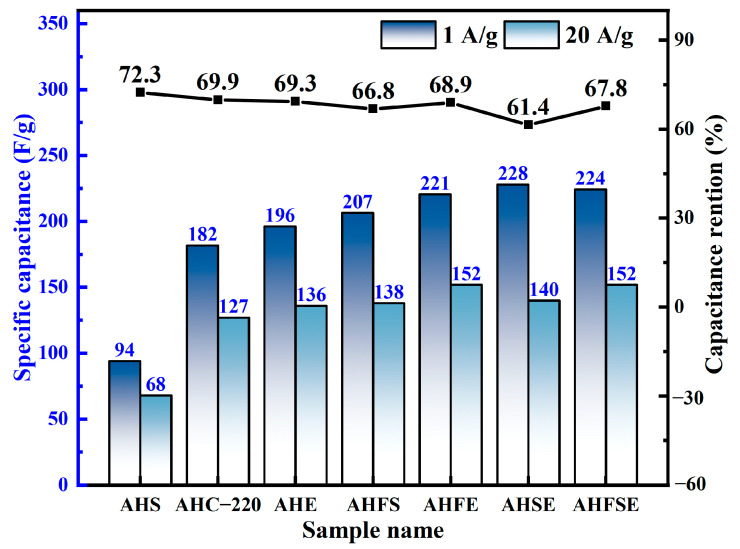
Specific capacitance of experimental samples of FW, SM, and EW hydrothermally doped at current densities of 1 A/g and 20 A/g and capacitance retention at high current densities.

As shown in Figure 8, the supercapacitor electrode material made from hydrothermally carbonized SM had the smallest specific capacitance, which was only 94.0 F/g. This suggests that the material’s electrochemical energy storage capacity was the lowest, but at high current densities, its capacitance retention rate was the highest. Compared with those of the carbon electrode materials produced solely through hydrothermal carbonization, the electrochemical energy storage capacity of the carbon electrode materials derived from the hydrothermal carbonization of waste doped with SM and EW in combination exhibited a notable increase. This suggests a positive synergistic effect between FW, SM, and EW during the hydrothermal carbonization process, leading to an improved electrochemical performance of the carbon electrode materials in the doping experiments.

#### 3.4.3. Electrochemical Performance of AMFSE

**Figure 9:**
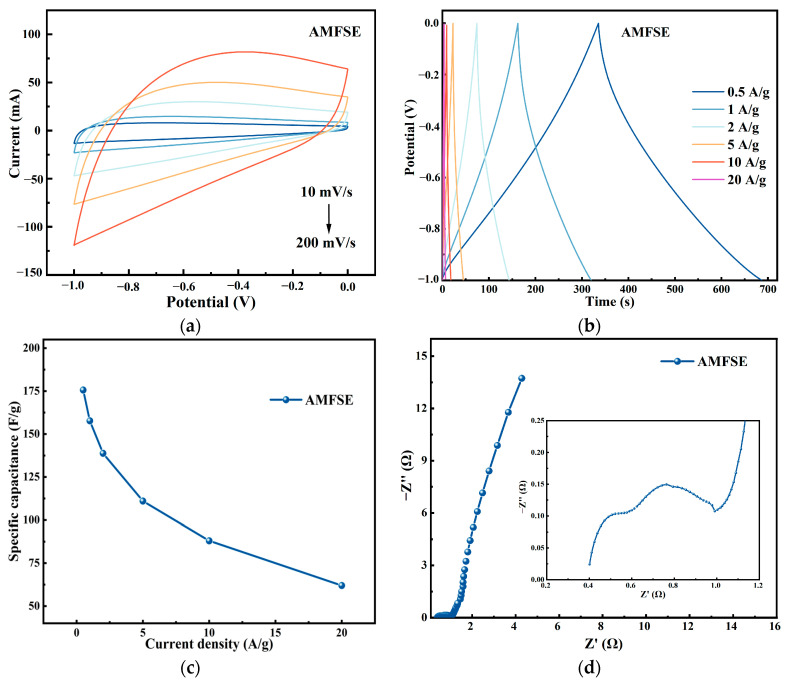
Electrochemical properties of AMFSE. (**a**) CV curves obtained at different current densities. (**b**) GCD curves (obtained at different current densities). (**c**) Specific capacitance curves obtained at different current densities. (**d**) Nyquist impedance plots.

To examine the variations in the electrochemical performance of carbon electrode materials for supercapacitors prepared by mutual doping between low-temperature pyrolytic carbonization and the hydrothermal carbonization of multiple samples, the electrochemical performance of sample AMFSE, which was obtained through the microwave pyrolysis of FW, SM, and EW, was also tested in a three-electrode system. As shown in Figure 9, the electrochemical performance results of the AMFSE sample were assessed via CV, GCD, and EIS tests. The AMFSE electrode CV curves at various scan rates are displayed in Figure 9a. The average electrochemical characteristics of the pyrolytic carbon created by microwave pyrolysis are indicated by the curves’ integrated area, which is significantly lower than that of the CV curves of AHFSE prepared by hydrothermal carbonization. The shuttle-shaped CV curve of the AMFSE sample suggested that its bilayer capacitance behavior was subpar. The primary cause of the shuttle-shaped CV curve was the inability of the electrolyte to swiftly penetrate the interior of the electrode material during the CV test. As a result, the polarization phenomenon occurred because the redox rate of the active material was lower than the rate at which electrons moved through the electrode. The enormous impedance of the electrode material is the primary cause of this problem. Figure 9b shows the GCD curve of the AMFSE electrode, showing a rapid voltage decrease at the beginning of discharge, which is also due to the large impedance of this electrode, resulting in a cliff-like decrease in the GCD curve at the beginning of discharge, which is consistent with its CV curve. Compared with that of the AHFSE sample at the same current density, the long-term energy storage capacity after one cycle of charging and discharging is less than that of the AHFSE sample, indicating its poor electrochemical energy storage capacity. Because this electrode’s electrochemical energy storage capacity is less than that of the AHFSE electrode, the specific capacitance curve of the AMFSE electrode in Figure 9c is significantly less than that of the AHFSE electrode in Figure 7c at the same current density. At 1 A/g, the AMFSE electrode’s specific capacitance is 157.7 F/g. Figure 9d shows the EIS data of the AMFSE sample. At high frequencies, the impedance curve is not a standard semicircular curve, and the impedance is large, mainly because pyrolysis cannot react between multiple materials and cause them to fuse together. The SEM results and the specific surface area test results show that the surface morphology of the sample is highly variable and that the surface has a square groove structure without an abundant microporous structure. The specific surface area is particularly small at 11.8 m^2^/g, which cannot provide sufficient loading medium for the active material; this is the main reason why the electrochemical performance of the AMFSE electrode is far inferior to that of the AHFSE electrode.

## 4. Conclusions

The XPS spectra of the activated doped carbon generated through the mutual doping carbonization of the FW with SM and EW showed a significantly elevated intensity of the N1s spectral peak, suggesting that the FW doped with SM and EW served a useful purpose in doping N. Additionally, the electrochemical performance was greatly enhanced, and the produced electrode materials demonstrated good bilayer capacitive behavior and electrochemical reversibility. At 1 A/g, the specific capacitance of the doped carbon largely surpassed 220.0 F/g, resulting in a significant improvement in the electrochemical energy storage capacity of the material. The specific capacitance of the AHFSE electrode was 224.0 F/g at a current density of 1 A/g; at 20 A/g, it remained at 152.0 F/g, and its capacitance retention at high potential was 67.86%. The primary reason for the discrepancy between the electrochemical performances of the AMFSE and AHFSE electrodes was that SM had a solid carbon skeleton structure and was less prone to pyrolysis, whereas EW was more susceptible to pyrolysis. However, EW was more prone to pyrolysis, with a stable carbon skeleton structure, large structural differences, and an uneven pore size distribution on the surface of the prepared electrode material resulting from microwave pyrolysis and KOH etching. This significantly reduced the ability of the material to transfer electrons during charging and discharging, increasing its impedance and making it unable to serve as an excellent loading carrier for the active material, both of which had a negative impact on the material’s electrochemical performance. To prepare materials for supercapacitor electrodes by interloping several samples, the hydrothermal carbonization procedure performed better than the microwave pyrolysis carbonization process. The main contribution of this research is the conversion of food waste into high-value-added carbon storage materials at lower temperatures through hydrothermal carbonization technology.

## Figures and Tables

**Table 1:** Elemental compositions of doped carbon and activated doped carbon.

Samples Name	C (%)	H (%)	N (%)	S (%)	O (%)
FW	48.17	7.04	2.72	0.19	41.02
SM	45.51	6.44	7.69	0.35	38.90
EW	44.61	6.97	3.98	0.43	43.38
HFS	64.10	7.17	6.74	0.37	21.30
HFE	66.38	6.42	5.42	0.43	21.21
HSE	64.54	6.71	7.54	0.45	20.53
HFSE	64.40	6.56	6.79	0.15	21.49
AHFS	77.30	1.58	6.59	0.23	14.06
AHFE	78.27	1.46	5.44	0.31	14.08
AHSE	79.13	2.11	5.29	0.22	12.68
AHFSE	77.58	1.66	5.67	0.19	14.41
AMFSE	74.69	1.43	5.05	0.20	17.98

**Table 2:** Results of the BET tests conducted on four different porous materials.

Samples Name	S_BET_ (m^2^/g)	S_micro_ (m^2^/g)	V_total_ (cm^3^/g)	V_micro_ (cm^3^/g)	D_pores_ (nm)
AHFE	207.8	179.8	0.11	0.09	2.20
AMFSE	11.8	2.95	0.03	0.0012	26.7
AHFSE	268.6	248.2	0.14	0.12	2.38
AHFS	22.4	4.42	0.07	0.0015	21.8

## Data Availability

The datasets used or analyzed during the current study are available from the corresponding author upon reasonable request.
